# Supplementary material for: Factors associated with neonatal pneumonia in India: protocol for a systematic review and planned meta-analysis
Source: BMJ Open. 2018 Jan 10;8(1):e018790. doi: 10.1136/bmjopen-2017-018790 (PMC5781011; doi:10.1136/bmjopen-2017-018790)
Supplement: Supplementary file [file bmjopen-2017-018790supp001.pdf]

DRAFT DATA EXTRACTION FORM

Section 1: General Information	Serial No	
	Date of extraction	*dd/mm/yyyy*
	Name of review author completing this form	*First name/middle name/ family name*
	Date form completed	*dd/mm/yyyy*
	Name of review author checking the data extracted to this form	*First name/middle name/ family name*
	Name of review author checking the data extracted to this form	*First name/middle name/ family name*
	Citation	*Vancouver style*
	URL	
	Other information and notes	
	Study ID	
Section 2: ELIGIBILITY	Study is reported in the Indian context?	*Yes/ No*
	Study is of observational design?	*Yes/ No*
	Study reports risk factor quantitative analysis for pneumonia in neonates?	*Yes/ No*
	Is study eligible for inclusion?	*Yes/ No*
Section 3: AUTHOR INFORMATION	Corresponding Author contact details for study	*Name*
		Affiliation
		Email id
		Phone number (if available)
	Further information required	
	Correspondence with authors successful or not; what information was received and when	
	Notes (Unpublished – for own use)	
Section 4: METHODS AND METHODOLOGY	Aim of study (as reported)	
	Study Design	
	Geographic location (s)	
	Setting (s)	*Hospital/ community*
		rural/ urban/ semi-urban
	Study Duration	
	Start date/ year	
	End date/ year	
	Number of groups	
	Inclusion criteria (for each group)	
	Exclusion criteria (for each group)	
	Operational definition of pneumonia used for study	
	Number in each group	
	Total subjects	
	Non-respondents	
	Duration of participation	*Recruitment*
		Data collection
		Follow up

	Method of recruitment of participants (e.g. phone, mail, clinic)	
	Data collection method	
	Source(s) of data for each group	
	Assumptions made	
	Variables included	*Independent*
		Dependant
	Confounding factors accounted for	*Confounding*
	Statistical methods	*Software*
		Tests
	Ethical approval obtained?	
	Informed consent obtained?	
	Sampling Technique	
	Sampling strategy	
	Sample size calculation:	
	Sample size	
	Eligible for inclusion	
	Excluded	
	Refused to take part	
	Cases/study cohort	
	Controls	
	Excluded (for each group; with reasons if relevant)	
	Withdrawn (for each group; with reasons if relevant)	
	Lost to follow up (for each group; with reasons)	
	Included in the analysis (for each group, for each outcome)	
**Section 5: RESULTS**	Population characteristics	*Age (years)*
		Gender
		Educational
		Income
		Socioeconomic strata
	Incidence/ Prevalence	*Number*
		Proportion
		other
		time to onset
	Risk factor	
	Group A events	
	Group A total	
	Group A rate and estimate	
	p	
	Confidence Interval (CI)	*lower*
		upper
	Group B events	
	Group B total	
	Group B rate and estimate	

	p	
	Confidence Interval (CI)	*lower*
		upper
	Group A Mean	
	Group A SD	
	Group B Mean	
	Group B SD	
	p	
	CI	*lower*
		upper
	Other findings	
	Conclusions	
	Limitations	
	Funding	
