# Supplementary material for: Factors associated with neonatal pneumonia in India: protocol for a systematic review and planned meta-analysis
Source: BMJ Open. 2018 Jan 10;8(1):e018790. doi: 10.1136/bmjopen-2017-018790 (PMC5781011; doi:10.1136/bmjopen-2017-018790)
Supplement: Author's manuscript [file bmjopen-2017-018790.draft_revisions.pdf]

BMJ Open is committed to open peer review. As part of this commitment we make the peer review history of every article we publish publicly available.

When an article is published we post the peer reviewers' comments and the authors' responses online. We also post the versions of the paper that were used during peer review. These are the versions that the peer review comments apply to.

The versions of the paper that follow are the versions that were submitted during the peer review process. They are not the versions of record or the final published versions. They should not be cited or distributed as the published version of this manuscript.

BMJ Open is an open access journal and the full, final, typeset and author-corrected version of record of the manuscript is available on our site with no access controls, subscription charges or pay-per-view fees (http://bmjopen.bmj.com).

If you have any questions on BMJ Open's open peer review process please email editorial.bmjopen@bmj.com

Factors associated with neonatal pneumonia in India: protocol for a systematic review and planned meta-analysis

Journal:	*BMJ Open*
Manuscript ID	bmjopen-2017-018790
Article Type:	Protocol
Date Submitted by the Author:	20-Jul-2017
Complete List of Authors:	Nair, N. ; j, Biostatistics<br>Lewis, Leslie; Manipal University Kasturba Medical College Manipal, paediatrics<br>Godinho, Myron ; Manipal University, Public Health Evidence South Asia (PHESA)<br>Murthy, Shruti; Manipal University, Public Health Evidence South Asia (PHESA)<br>Lakiang, Theophilus; Manipal University, Public Health<br>Venkatesh , Bhumika; Manipal University
**Primary Subject Heading**:	Paediatrics
Secondary Subject Heading:	Infectious diseases, Paediatrics
Keywords:	NEONATOLOGY, Neonate, Pneumonia, Risk factor, Systematic Review, Meta-analysis

SCHOLARONE™
Manuscripts

For peer review only - http://bmjopen.bmj.com/site/about/guidelines.xhtml

Factors associated with neonatal pneumonia in India: protocol for a systematic review and planned meta-analysis

N. Sreekumaran Nair[1], Leslie Edward Lewis[2], Myron Godinho[3*], Shruti Murthy [3**], Theophilus Lakiang[3***], Bhumika T. Venkatesh[4]

[1] Director, Public Health Evidence South Asia (PHESA). nsknairmanipal@gmail.com

[2] Professor and Head, Neonatology Unit, Department of Pediatrics, Kasturba Medical College, Manipal University, Manipal. leslie.lewis@manipal.edu

[3] Research Associate, Public Health Evidence South Asia (PHESA), Manipal University, Manipal;

*myrongodinho@gmail.com;

** itzshruti@gmail.com;

***theo.lakiang@gmail.com ;

[4]Assistant Professor, Public Health Evidence South Asia (PHESA), Manipal University, Manipal; meetbhumika123@gmail.com

Correspondence to:
Dr. N. Sreekumaran Nair
Director, Public Health Evidence South Asia (PHESA), Ground Floor,
Manipal EDU Building, Manipal University,
Manipal-576104, Karnataka, India
Email address: nsknairmanipal@gmail.com
Telephone number: +91-9900133734

PROSPERO 2016:CRD42016044019

For peer review only

Factors associated with neonatal pneumonia in India: protocol for a systematic review and planned meta-analysis

Abstract

Introduction: India contributes to the highest number of neonatal deaths globally. There is a lack of consolidated evidence from India regarding the risk factors of pneumonia in neonates. We aim to systematically review the evidence for risk factors of neonatal pneumonia in India.

Methods and analysis: This protocol is part of a series of three systematic reviews on neonatal pneumonia in India. Observational studies reporting on neonatal pneumonia in the Indian context, and published in English in peer-reviewed and indexed journals will be eligible for inclusion. Outcome of this review will be risk factors of neonatal pneumonia. A total of nine databases will be searched. Electronic and hand searching of published and grey literature will be performed. Selection of studies will be done in title, abstract and full text stages. Risk of bias, independently assessed by two authors, will be evaluated. Meta-analysis will be performed and heterogeneity assessed. Pooled effect estimates will be stated with 95% confidence intervals. Narrative synthesis will be done where meta-analysis cannot be performed. Publication bias will be evaluated and sensitivity analysis performed according to study quality. Quality of this review will be evaluated using AMSTAR (Assessing the Methodological quality of Systematic Reviews) and GRADE (Grades of Recommendation, Assessment, Development & Evaluation). A summary of findings table will be reported using GRADEPro.

Ethics and dissemination: Since this is a review involving analysis of secondary data which is available in the public domain, and does not involve human participants, ethical approval was not required. The findings of the study will be shared with all stakeholders of this research. Knowledge dissemination workshops will be conducted with relevant stakeholders to ultimately transfer the evidence tailored to the stakeholder (e.g. policy briefs, publications, information booklets, etc).

Keywords: Neonate, Pneumonia, Risk factors, Systematic Review, Meta-analysis, India

For peer review only - http://bmjopen.bmj.com/site/about/guidelines.xhtml

Strengths and limitations of the study:

- First review to consolidate evidence on risk factors of neonatal pneumonia in India.
- A comprehensive search strategy was developed over 9 databases including relevant regional databases and grey literature published in any year.
- We considered only English language studies and studies which assessed pneumonia independent of neonatal sepsis. We attempted to capture definition of neonatal pneumonia reported in each individual study.

Introduction

In 2015, all over the world, 5.9 million children died before their fifth birthday.[1] Twenty percent of these deaths occurred in India; more than in any other country.[1] In India, neonatal mortality accounts for 57% of national under-five deaths.[2] Additionally, India accounts for the highest global neonatal deaths amounting to 26% (696,000) .[3] The main causes for neonatal mortality In India are prematurity (35%), birth asphyxia & birth trauma (20%) and infectious diseases (33%).[2] Deaths due to infections are caused by pneumonia (16%), sepsis (15%), and diarrhoea (2%).[4] Neonatal pneumonia in India is clearly an issue of global importance.

Most studies have focused only on sepsis and pneumonia in children aged under-five. Neonates are considerably different from post-neonatal children, both in their physiology and the environment they are exposed to,[5] and are more affected by antenatal factors such as childbirth before 20 years of age, and spacing between successive births.[6] Consolidated evidence on factors determining neonatal pneumonia in India is greatly lacking, and urgently required. Hence the objective of this systematic review is to systematically review the evidence for risk factors and determinants associated with neonatal pneumonia in the Indian context.

Methods and analysis

This protocol is part of a trilogy of reviews on neonatal pneumonia in India and has been developed based on the 'Meta-analysis of Observational Studies in Epidemiology' (MOOSE) guidelines and Preferred Reporting Items for Systematic reviews and Meta-Analysis-Protocol' (PRISMA-P) guidelines.[7] This review will be conducted from August 2016 to

For peer review only - http://bmjopen.bmj.com/site/about/guidelines.xhtml

October 2017.

Criteria for considering studies for this review

Types of studies

Inclusion criteria: Published studies reporting on factors or determinants of neonatal pneumonia in the Indian context will be eligible for inclusion. To be eligible for inclusion, studies must be published in English language in indexed and peer-reviewed journals and must be of the following observational study designs including (a) analytic studies- case-control study; cohort study, analytical cross sectional study and (b) descriptive studies (case series, cross sectional studies), reports of secondary data analysis, outcome studies, fact sheets which report a quantitative analysis of risk factors of neonatal pneumonia.

Exclusion criteria: Intervention studies, all reviews, meta-analyses, qualitative research, letters, editorials, commentaries, conference papers, reports which do not include a quantitative analysis of risk factors of neonatal pneumonia will be excluded. Since neonatal pneumonia is often considered a component of neonatal sepsis, we will consider such studies for inclusion if the study specifically reports on the pneumonia component explicitly.

Type of participants**:** Neonates in the Indian context.

Outcome of the review**:** Outcomes of this review will be the risk factors and determinants of neonatal pneumonia. Definitions of 'risk factor' and 'determinant' reported by the authors will be captured in the review.

Search methods for identification of studies

An exhaustive list of search terms will be used to develop an appropriate and comprehensive search strategy for final searches.

Electronic searches**:** Electronic search will be done on nine databases: PubMed, Ovid MEDLINE, Web of Science, CINAHL, EMBASE, SCOPUS, IMSEAR, ProQuest and IndMED.

Hand searching**:** Hand searching will be conducted for volumes of journals not included in the electronic databases, and conference proceedings to review the references and contact the authors for full texts of identified literature.

Searching the grey literature**:** Potential sources of grey literature will include Shodhganga (INFLIBNET) and Government of India databases.

Reference lists**:** Snowballing will be done to screen references of identified literature for potentially relevant studies.

For peer review only - http://bmjopen.bmj.com/site/about/guidelines.xhtml

Moreover, authors, subject experts, researchers and relevant organizations of identified studies will be contacted to find other existing relevant studies.

An example of our search strategy is presented in Table 1.

Table 1. Search strategy (PubMED)

Strategy: #1 AND #2 AND #3	
#1	(((Neonate* OR childhood OR neonatal* OR newborn* OR "young infant" OR child OR pediatric* OR paediatric* OR "neonatal period" OR infant* OR "newborn infant"))
#2	((((((((((((((((((((((((((((((Pneumonia*) OR Pneumon*) OR "community acquired pneumonia") OR "congenital pneumonia") OR "hospital acquired pneumonia") OR "nosocomial pneumonia") OR "ventilator associated pneumonia") OR "early onset pneumonia") OR "late onset pneumonia") OR "infective pneumonia") OR "infectious pneumonia") OR "meconium aspiration syndrome") OR "meconium aspiration") OR "lipoid pneumonia") OR sepsis*) OR "acute respiratory infections") OR "early onset sepsis") OR "chemical pneumonia") OR "aspiration pneumonia") OR "late onset sepsis") OR infection*) OR "nosocomial infection") OR "early onset infection") OR "late onset infection") OR "acute lower respiratory infection") OR "hospital acquired infection") OR "congenital infection") OR "viral pneumonia") OR "gastro esophageal reflux disease") OR "cystic fibrosis")
#3	((("Risk factor" OR determinant* OR risk* OR predictor* OR "relative risk" OR "odds ratio" OR "attributable risk" OR "population attributable fraction"))))
Geographical Filter: India<br>Language Filter: English	

Data collection and management

The results (titles and/or abstracts) of the search will be managed using Endnote (v. x7). Study selection will be performed on Endnote (v. x7). Data will be extracted on Microsoft Excel 2007. Statistical analysis will be performed using STATA (v.13).

Selection of studies

Screening of search results for eligible studies will be performed by two authors (MG and SM) independently in title, abstract and full text stages. During the title screening stage,

For peer review only

For peer review only - http://bmjopen.bmj.com/site/about/guidelines.xhtml

titles approved by either author will proceed to abstract screening. If both authors reject a title at this stage, it will be excluded from the review. At the abstract screening stage, abstracts approved by either author will proceed to full text screening. If both authors reject an abstract at this stage, it will be excluded from the review. For the full text screening, only those studies approved by both authors will be included in the review. Any disagreements will be resolved through arbitration and consensus by a third author (TL) and senior review authors (SN and LL). Rationale for exclusion will be provided for all excluded studies. A final list of articles will be prepared for data extraction. A PRISMA chart will be generated to illustrate this study selection process.[8]

Data extraction

From the selected studies, data will be extracted with the use of a standardised, pre-tested data extraction form by two authors (MG and SM) independently. Any disagreements will be resolved by discussion and consensus between the authors. The data extraction form was developed by the authors, with technical expertise from senior reviewers, subject and clinical experts and statisticians. The form has been pilot-tested on one study of each type to ensure that it adequately facilitated the collection of all necessary information required for an effective meta-analysis. Data will be extracted under the following major domains (a) study characteristics (b) methodological characteristics (c) factors identified (factors, type of data, measure of association calculated) and (d) other important information.

Dealing with missing data

In instances of inadequacy, missing information, lack of clarity on information in methodology or outcomes, authors of the respective studies will be contacted in an attempt to obtain the required information. Despite this effort, if the missing data retrieval is not possible, the particular study will be included in the systematic review and discussed in the narrative summary. However, this study will be excluded from meta-analysis and analysis of only the available data will be performed.

Assessment of risk of bias in included studies

Risk of bias assessment will be done at the study level. The Newcastle-Ottawa Scale (NOS) will be used to assess the risk of bias in case-control and cohort studies.[9] An adapted NOS

For peer review only - http://bmjopen.bmj.com/site/about/guidelines.xhtml

will be used for the appraisal of cross-sectional studies.[10] The quality of case series will be assessed using the Institute of Health Economics (IHE) criteria.[11] Risk of bias assessment of the included studies will be performed by two authors (SM and TL) independently at both the study and outcome levels. Disagreements will be resolved by discussion and involvement of a third (MG) or senior authors (SN and LL), when necessary. The outcome of this assessment and its implications on the outcome of the meta-analysis will be discussed in the final narrative synopsis.

Data analysis

Data synthesis: A meta-analysis will be used to consolidate quantitative data. Data, as reported in the studies, will first be extracted. Next, if required, they will be transformed.

Categorical data: Categorical data will be summarized using measures such as odds ratio (OR) and relative risk (RR) for risk estimation.

Continuous data: Continuous data will be summarized using the standardized mean difference (SMD).

The summary measures will be pooled together according to the study design. Pooled effect estimates will be stated with 95% confidence intervals quantitatively and as a forest plot.[12]

A narrative summary will describe factors for which a meta-analysis is impossible; (reason for exclusion from meta-analysis will be provided).

Investigation of heterogeneity: A fixed-effects or a random-effects model will be employed, based on study heterogeneity, determined by calculating an I^2 statistic with 95% confidence intervals. Possible reasons for heterogeneity will be discussed.

Sub group analysis: Subgroup analysis will be done, when data are available, to compare risk factors according to, but not limited to, (a) study design (b) type of neonatal pneumonia (c) setting and (d) timing of onset. The final sub grouping will be decided after data extraction.

Meta-regression: Meta-regression will also be conducted in order to determine the effect of the covariates on the pooled effect size, and allow for adjustment of this effect to account for heterogeneity due to varying contexts and populations

Sensitivity analysis: Sensitivity analysis will be provided based on study quality; the change in the pooled effect result will be measured to determine the role of the relevant study.

For peer review only - http://bmjopen.bmj.com/site/about/guidelines.xhtml

For peer review only

Assessment of reporting bias: To assess the publication bias, a funnel plot will be generated and Egger's test performed to assess the degree of asymmetry if there are more than 10 studies included in the review.

Quality control of the systematic review and meta-analysis: A Measurement Tool to Assess Systematic Reviews (AMSTAR) criteria will be used to evaluate the methodological quality of the systematic review.[13] The Grading of Recommendations Assessment, Development and Evaluation (GRADE) assessment will be performed to evaluate the quality of evidence generated, and a summary of findings table will be reported using GRADEPro.

Ethics and dissemination:

Ethics: Since this is a review involving analysis of secondary data which is available in the public domain, and does not involve human participants, ethical approval was not required.

Dissemination: The findings of the study will be shared with all stakeholders of this research. Knowledge dissemination workshops will be conducted with relevant stakeholders to ultimately transfer the evidence tailored to the stakeholder (e.g. policy briefs, publications, information booklets, etc).

Reporting of the systematic review and meta-analysis: The findings of this systematic review will be reported in accordance with the PRISMA Guidelines and the MOOSE Guidelines.[14],[8]

Acknowledgements

We would like to thank the following individuals for their continuous support and guidance during this process of protocol development: Dr. Manoj Das, Director Projects, The INCLEN Trust International, New Delhi; Dr Anju Sinha, Deputy Director General, Scientist 'E', Division of Child Health, Indian Council of Medical Research, New Delhi; Dr. K.K. Diwakar, Professor and Head, Department of Neonatology, Associate Dean, Malankara Orthodox Syrian Church Medical College, Kerala; Mrs. Ratheebhai V., Senior Librarian and Information Scientist, at Manipal School at Communication, Manipal University, Manipal; Dr. Ravinder M. Pandey, Professor and Head, Department of Biostatistics, All India Institute of Medical Sciences, New

For peer review only - http://bmjopen.bmj.com/site/about/guidelines.xhtml

Delhi; Dr. B. Shantharam Baliga, Professor, Department of Paediatrics, Kasturba medical college, Mangalore, Karnataka, ; Dr. Shirish Darak, Senior researcher, PRAYAS, Pune, Maharashtra; Dr. Unnikrishnan B., Associate Dean and Professor, Department of Community Medicine, Kasturba Medical College, Mangalore. We also thank Public Health Evidence South Asia (PHESA) and Manipal University, Manipal for providing the necessary institutional and infrastructural support for the project. We would also like to thank The INCLEN Trust International, New Delhi, and The Bill and Melinda Gates Foundation for the financial support which made this project possible.

Contributorship statement: SN is the guarantor of the review. SN, BV and LL conceived the research idea and reviewed the manuscript. SN and LL provided overall technical guidance. In addition, LL assisted in developing search terms. MG, SM and TL designed the protocol, drafted the manuscript, and developed and pilot tested the search strategies and data extraction form.

Funding: This project is supported by a grant from Bill & Melinda Gates Foundation [grant OPP1084307] to The INCLEN Trust International and sub-grant to Manipal University [subgrant INC2015GNT004). The views expressed through this project do not necessarily represent the views of Bill and Melinda Gates Foundation or The INCLEN Trust International or Manipal University.

Data sharing statement: All data supporting this study will be provided as supplementary material together with the manuscript of the study's final results.

Competing Interests: All authors have completed the ICMJE uniform disclosure form at www.icmje.org/coi_disclosure.pdf and declare: all authors had financial support (grants) from Bill and Melinda Gates Foundation (grant OPP1084307) to The INCLEN Trust International and sub-grant to Manipal University (subgrant INC2015GNT004)., during the conduct of the study and for the submitted work; no financial relationships with any organisations that might have an interest in the submitted work in the previous three years; no other relationships or activities that could appear to have influenced the submitted work.

References

For peer review only - http://bmjopen.bmj.com/site/about/guidelines.xhtml

1. UNICEF. Committing to Child Survival: A Promise Renewed, Progress Report 2015. 2015. *New York, USA: UNICEF* September 2015
2. Department of Health & Family Welfare MoHFW, Government of India. Ch 5: Child Health Program, Annual Report 2014-15 2015 [cited 2016 18/1/2016]. Available from: http://www.mohfw.nic.in/WriteReadData/l892s/5658789632541236.pdf.
3. You D, Hug L, Ejdemyr S, et al. Levels and trends in child mortality. Report 2015. Estimates developed by the UN Inter-agency Group for Child Mortality Estimation. 2015
4. World Health Organisation (WHO). Neonatal and Child Health Profile, India: Department of Maternal Newborn Child and Adolescent Health 2013 [cited 2016 19/1/2016]. Available from: http://www.who.int/maternal_child_adolescent/epidemiology/profiles/neonatal_child/ind.pdf.
5. Public Health Foundation of India (PHFI) A. SC-State of India's Newborns (SOIN) 2014 - a report. *Public Health Foundation of India, All India Institute of Medical Sciences and Save the Children New Delhi, India Available at http://wwwnewbornwhoccorg/SOIN_PRINTED* 2014:9-2014.
6. United Nations Children's Fund (UNICEF) and National Institute of Medical Statistics, Indian Council of Medical Research (ICMR). Infant and Child mortality in India: Levels, trends and determinants (Fact sheet) [cited 2016 3/9/2016]. Available from: http://unicef.in/CkEditor/ck_Uploaded_Images/img_1364.pdf.
7. Shamseer L, Moher D, Clarke M, et al. Preferred reporting items for systematic review and meta-analysis protocols (PRISMA-P) 2015: elaboration and explanation. *Bmj* 2015;349:g7647.
8. Liberati A, Altman DG, Tetzlaff J, et al. The PRISMA statement for reporting systematic reviews and meta-analyses of studies that evaluate health care interventions: explanation and elaboration. *Annals of internal medicine* 2009;151(4):W-65-W-94.
9. Wells G, Shea B, O'connell D, et al. The Newcastle-Ottawa Scale (NOS) for assessing the quality of nonrandomised studies in meta-analyses, 2000.
10. Herzog R, Álvarez-Pasquin MJ, Díaz C, et al. Are healthcare workers' intentions to vaccinate related to their knowledge, beliefs and attitudes? A systematic review. *BMC public health Available at: http://static-contentspringercom/esm/art%3A101186%2F1471-2458-13-154/MediaObjects/12889_2012_5111_MOESM3_ESMdoc* 2013;13(1):1.
11. Moga C, Guo B, Schopflocher D, et al. Development of a quality appraisal tool for case series studies using a modified Delphi technique. Institute of Health Economics. Alberta Canada, 2012.
12. DerSimonian R, Laird N. Meta-analysis in clinical trials revisited. *Contemporary clinical trials* 2015;45:139-45.
13. Shea BJ, Grimshaw JM, Wells GA, et al. Development of AMSTAR: a measurement tool to assess the methodological quality of systematic reviews. *BMC medical research methodology* 2007;7(1):1.
14. Stroup DF, Berlin JA, Morton SC, et al. Meta-analysis of observational studies in epidemiology: a proposal for reporting. *Jama* 2000;283(15):2008-12.

For peer review only

For peer review only - http://bmjopen.bmj.com/site/about/guidelines.xhtml

PRISMA-P (Preferred Reporting Items for Systematic review and Meta-Analysis Protocols) 2015 checklist: recommended items to address in a systematic review protocol*

Section and topic	Item No	Checklist item	Compliance
**ADMINISTRATIVE INFORMATION**			
Title:			
Identification	1a	Identify the report as a protocol of a systematic review	Pg 1& 2
Update	1b	If the protocol is for an update of a previous systematic review, identify as such	Not applicable
Registration	2	If registered, provide the name of the registry (such as PROSPERO) and registration number	Pg 1
Authors:			
Contact	3a	Provide name, institutional affiliation, e-mail address of all protocol authors; provide physical mailing address of corresponding author	Pg 1
Contributions	3b	Describe contributions of protocol authors and identify the guarantor of the review	Pg 9
Amendments	4	If the protocol represents an amendment of a previously completed or published protocol, identify as such and list changes; otherwise, state plan for documenting important protocol amendments	Not applicable
Support:			
Sources	5a	Indicate sources of financial or other support for the review	Pg 9
Sponsor	5b	Provide name for the review funder and/or sponsor	Pg 9
Role of sponsor or funder	5c	Describe roles of funder(s), sponsor(s), and/or institution(s), if any, in developing the protocol	Pg 9
**INTRODUCTION**			
Rationale	6	Describe the rationale for the review in the context of what is already known	Pg 3
Objectives	7	Provide an explicit statement of the question(s) the review will address with reference to participants, interventions, comparators, and outcomes (PICO)	Pg 3 Modified accordingly for review of non-interventional studies
**METHODS**			
Eligibility criteria	8	Specify the study characteristics (such as PICO, study design, setting, time frame) and report characteristics (such as years considered, language, publication status) to be used as criteria for eligibility for the review	Pg 4 Modified accordingly for

For peer review only

			review of non-interventional studies
Information sources	9	Describe all intended information sources (such as electronic databases, contact with study authors, trial registers or other grey literature sources) with planned dates of coverage	Pg 4-5
Search strategy	10	Present draft of search strategy to be used for at least one electronic database, including planned limits, such that it could be repeated	Pg 5
Study records:			
Data management	11a	Describe the mechanism(s) that will be used to manage records and data throughout the review	Pg 5
Selection process	11b	State the process that will be used for selecting studies (such as two independent reviewers) through each phase of the review (that is, screening, eligibility and inclusion in meta-analysis)	Pg 5-6
Data collection process	11c	Describe planned method of extracting data from reports (such as piloting forms, done independently, in duplicate), any processes for obtaining and confirming data from investigators	Pg 6
Data items	12	List and define all variables for which data will be sought (such as PICO items, funding sources), any pre-planned data assumptions and simplifications	Pg 6
Outcomes and prioritization	13	List and define all outcomes for which data will be sought, including prioritization of main and additional outcomes, with rationale	Pg 7
Risk of bias in individual studies	14	Describe anticipated methods for assessing risk of bias of individual studies, including whether this will be done at the outcome or study level, or both; state how this information will be used in data synthesis	Pg 6-7
Data synthesis	15a	Describe criteria under which study data will be quantitatively synthesised	Pg 7
	15b	If data are appropriate for quantitative synthesis, describe planned summary measures, methods of handling data and methods of combining data from studies, including any planned exploration of consistency (such as I^2, Kendall's τ)	Pg 7
	15c	Describe any proposed additional analyses (such as sensitivity or subgroup analyses, meta-regression)	Pg 7
	15d	If quantitative synthesis is not appropriate, describe the type of summary planned	Pg 7
Meta-bias(es)	16	Specify any planned assessment of meta-bias(es) (such as publication bias across studies, selective reporting within studies)	Pg 7-8
Confidence in cumulative evidence	17	Describe how the strength of the body of evidence will be assessed (such as GRADE)	Page 8

*** It is strongly recommended that this checklist be read in conjunction with the PRISMA-P Explanation and Elaboration (cite when available) for important clarification on the items. Amendments to a review protocol should be tracked and dated.** **The copyright for PRISMA-P (including checklist) is held by the PRISMA-P Group and is distributed under a Creative Commons Attribution Licence 4.0.**

From: Shamseer L, Moher D, Clarke M, Ghersi D, Liberati A, Petticrew M, Shekelle P, Stewart L, PRISMA-P Group. Preferred reporting items for systematic review and meta-analysis protocols (PRISMA-P) 2015: elaboration and explanation. BMJ. 2015 Jan 2;349(jan02 1):g7647.

For peer review only

BMJ Open

Factors associated with neonatal pneumonia in India: protocol for a systematic review and planned meta-analysis

Journal:	*BMJ Open*
Manuscript ID	bmjopen-2017-018790.R1
Article Type:	Protocol
Date Submitted by the Author:	17-Oct-2017
Complete List of Authors:	Nair, N. ; j, Biostatistics<br>Lewis, Leslie; Manipal University Kasturba Medical College Manipal, paediatrics<br>Godinho, Myron ; Manipal University, Public Health Evidence South Asia (PHESA)<br>Murthy, Shruti; Manipal University, Public Health Evidence South Asia (PHESA)<br>Lakiang, Theophilus; Manipal University, Public Health<br>Venkatesh , Bhumika; Manipal University
**Primary Subject Heading**:	Infectious diseases
Secondary Subject Heading:	Paediatrics, Public health, Epidemiology
Keywords:	NEONATOLOGY, Neonatal intensive & critical care < INTENSIVE & CRITICAL CARE, Epidemiology < INFECTIOUS DISEASES, Paediatric infectious disease & immunisation < PAEDIATRICS, Systematic Review, Pneumonia

SCHOLARONE™
Manuscripts

Factors associated with neonatal pneumonia in India: protocol for a systematic review and planned meta-analysis

N. Sreekumaran Nair[1], Leslie Edward Lewis[2], Myron Godinho[3*], Shruti Murthy [3**], Theophilus Lakiang[3***], Bhumika T. Venkatesh[4]

[1] Director, Public Health Evidence South Asia (PHESA). nsknairmanipal@gmail.com

[2] Professor and Head, Neonatology Unit, Department of Pediatrics, Kasturba Medical College, Manipal University, Manipal. leslie.lewis@manipal.edu

[3] Research Associate, Public Health Evidence South Asia (PHESA), Manipal University, Manipal;

*myrongodinho@gmail.com;

** itzshruti@gmail.com;

***theo.lakiang@gmail.com ;

[4]Assistant Professor, Public Health Evidence South Asia (PHESA), Manipal University, Manipal; meetbhumika123@gmail.com

Correspondence to:
Dr. N. Sreekumaran Nair
Director, Public Health Evidence South Asia (PHESA), Ground Floor,
Manipal EDU Building, Manipal University,
Manipal-576104, Karnataka, India
Email address: nsknairmanipal@gmail.com
Telephone number: +91-9900133734
PROSPERO 2016:CRD42016044019

For peer review only

Factors associated with neonatal pneumonia in India: protocol for a systematic review and planned meta-analysis

Abstract

Introduction: India accounts for more neonatal deaths than any other country. There is a lack of consolidated evidence from India regarding the determining factors of pneumonia in neonates. This systematic review is aimed to consolidate and appraise the evidence on risk factors and determinants of pneumonia among neonates in India.

Methods and analysis: This protocol is part of a project consisting of three reviews (two systematic reviews and one scoping review) and a qualitative study on neonatal pneumonia in India. English language observational studies which report risk factors and determinants of neonatal pneumonia in India will be eligible for inclusion. Electronic searching of nine databases, and hand searching will be done. Two authors will independently conduct screening (title, abstract and full text stages), extract data and assess risk of bias. A meta-analysis is planned to be performed with random-effects model. A narrative synthesis will be used to summarize the characteristics and findings of the review, if a meta-analysis cannot be performed. If there are more than ten studies, publication bias will be assessed. Sensitivity and subgroup analysis will performed based on data availability. The quality of our review will be assessed by using “Assessing the Methodological quality of Systematic Reviews” (AMSTAR) and “Grades of Recommendation, Assessment, Development & Evaluation” (GRADE).

Ethics and dissemination: The protocol of the entire project has been approved by the host institution’s ethics body, (Institutional Ethics Committee, Manipal University in Manipal at India), and the ‘Health Ministry Screening Committee’ (HMSC) under the Ministry of Health and Family Welfare, Government of India. The study findings will be disseminated among relevant stakeholders using knowledge dissemination workshops, policy briefs, publications, etc.

Keywords: Neonate, Pneumonia, Risk factors, Systematic Review, Meta-analysis, India

Strengths and limitations of the study:

For peer review only - http://bmjopen.bmj.com/site/about/guidelines.xhtml

For peer review only

- This is the first systematic review and meta-analysis on risk factors of pneumonia among neonates in India.
- Use of a sensitive search strategy over nine databases, including regional databases and websites. Both published and grey literature will be searched.
- No time restriction will be applied for searches, and inclusion.
- We will consider studies which reported on pneumonia among neonates independent of sepsis, and that published in English. We will capture the definitions of neonatal pneumonia as reported in included studies.

Introduction

Globally, nearly 5 million children aged under five died in 2016; regionally, South Asia contributed nearly a quarter (24.8%) of the global burden of under-5 mortality, with almost a million deaths occurring in India alone, the largest of any nation.[1] In 2016, the global neonatal mortality rate (NMR) of 16.7 deaths per 1000 live births exceeded both post neonatal mortality (11.7 per 1000 livebirths) and mortality in the 1-4 year age group (10.5 per 1000 livebirths). The South Asian NMR was estimated at 23.2 deaths per 1000 livebirths, with India's NMR of 21.8 deaths per 1000 live births being the second highest in the region, only exceeded by Pakistan (31.8 per 1000 live births).[1]

In India, neonatal mortality accounts for 57% of national under-five deaths.[2]

33% of neonatal mortality in India is due to infectious disease,[2] comprising pneumonia (16%), sepsis (15%), and diarrhoea (2%).[3] Pneumonia in neonates contributes significantly to mortality in infants, with over half of childhood pneumonia deaths occuring during the newborn period.[4]

Most studies have focused only on sepsis and pneumonia in children aged under-five. Neonates are considerably different from post-neonatal children, both in their physiology and the environments they are exposed to,[5] and are more affected by antenatal factors (e.g. teenage pregnancy, poor spacing between successive births, etc.)[6] Neonatal pneumonia is clinically categorized as 'early onset' or 'late onset' - each of which determined by factors in the intra-uterine or the external environment, respectively. [7] Different arrays of pathogens produce different variants of neonatal pneumonia.[8] Early-onset neonatal pneumonia is

For peer review only - http://bmjopen.bmj.com/site/about/guidelines.xhtml
For peer review only

often due to pathogens aspirated by the neonate from the intrauterine environment, or from the birth canal during vaginal delivery. The risk is particularly high if the mother has chorioamnionitis - an infection of the intra-uterine tissues.[9] Late-onset pneumonia is often caused by pathogens encountered in the post-natal environment, either in the community (Community-associated pneumonia, CAP), or in the hospital (Hospital-associated pneumonia, HAP). Neonates with breathing difficulties (commonly due to prematurity or hyaline membrane disease) often require mechanical ventilation through an endotracheal tube, which can serve as a route for introducing nosocomial pathogens, causing ventilator-associated pneumonia (VAP).[10] Pneumonia can be experienced by neonates who have dysphagia (difficulty swallowing) due to neuromuscular disorders, anomalies of the oropharyngeal anatomy (e.g cleft palate, tracheo-oesophageal fistulae), or who experience gastro-esophagaeal reflux disease (GERD). Lipoid pneumonia is due to aspiration of oil, and is not unusual in India due to the regional sociocultural practice of bathing newborn children in oil or ghee, and cleaning their nose and mouth with the same.[11] In India, poverty, malnutrition, impaired access to healthcare, poor immunization status, poor child-rearing practices, and indoor air pollution, are common risk factors for pneumonia.[9 12]

Consolidated evidence on the factors responsible for the Indian burden of neonatal pneumonia is greatly lacking and urgently required. This protocol aims to describe the methods that will be used to conduct a systematic of the evidence on risk factors and determinants of with neonatal pneumonia in the Indian context. With this study, we propose to identify various risk factors and determinants associated with neonatal pneumonia, in order to better understand, identify and eliminate these risk factors and determinants, and thus reduce the disease burden of neonatal pneumonia in India.

This protocol is part of a project consisting of three reviews (two systematic reviews[13] and one scoping review[14]) and a qualitative study[15] on neonatal pneumonia in India. The other systematic review and planned meta-analysis will consolidate and appraise the evidence relating to the predictors of mortality due to neonatal pneumonia in India, and has been described elsewhere.[13] The scoping review will identify and summarize the evidence on the barriers to pneumonia case management and options for treatment.[14] In addition to the three reviews, a qualitative study will attempt to capture perceptions of stakeholders in the neonatal healthcare system.[15]

For peer review only - http://bmjopen.bmj.com/site/about/guidelines.xhtml
For peer review only

Methods and analysis

This protocol is developed and reported based on the "Meta-analysis of Observational Studies in Epidemiology" (MOOSE) guidelines,[16] and "Preferred Reporting Items for Systematic reviews and Meta-Analysis- Protocol" (PRISMA-P) guidelines.[17] This systematic review and meta-analysis will be conducted from August 2016 to January 2018.

Criteria for considering studies for this review

Types of studies

Inclusion criteria: Observational studies, published in English, which report on risk factors or determinants of pneumonia among neonates in India will be considered eligible for inclusion. Studies can be either descriptive (e.g. case series, cross-sectional design) or analytical in design (e.g. case-control, cohort, analytical cross-sectional designs). Reports of secondary data analysis and fact sheets should provide risk factor estimates to be considered for inclusion in the review. Studies published in English language will be eligible for inclusion. It is unlikely that languages other than English would be the primary language in which the evidence reports would be published. Attempts to identify relevant material published in other languages will be made by contacting established networks and experts in the review topic to suggest any studies in local language on the topic.

Exclusion criteria: Intervention studies, all reviews, meta-analyses, qualitative research, conference abstracts/papers, letters, editorials, commentaries, , reports which do not provide risk factor estimates for neonatal pneumonia will be excluded. Since neonatal pneumonia is often considered a component of neonatal sepsis, we will consider such studies for inclusion if the study specifically reports on the pneumonia component explicitly.

Type of participants**:** Neonates in India.

Outcome of the review include risk factors and determinants of neonatal pneumonia. Definitions of 'risk factor' and 'determinant' as reported by the included studies will be captured in our review. Examples of operational definitions of these key terms are as follows:

For peer review only

Risk factor: “A risk factor is any attribute, characteristic or exposure of an individual that increases the likelihood of developing a disease or injury (or other negative/undesirable outcomes)”.[18]

Health determinant: “Underlying personal/social/economic/environmental characteristics which ultimately shape the health of individuals and communities. They are ‘upstream factors’ or, the causes of the causes of ill health”.[19]

Table 1 outlines possible (not exhaustive) risk factors for pneumonia among neonates, which have been adapted through consultation with subject experts and from existing literature.[20,21,22,23] This list will be modified based on the findings from this systematic review.

Table 1: Possible risk factors of neonatal pneumonia

Category	Factors
Patient-related	Age<br>Gender<br>Low Birth Weight<br>Small-for-age<br>Prematurity<br>Birth order<br>APGAR score at birth<br>Concomitant conditions/comorbid illnesses (e.g congenital heart disease, asthma, hemoglobin, cyanosis, hypothermia)<br>Congenital malformations<br>Feeding practices<br>Others
Parent-related	Socio-demographics<br>Economic<br>Cultural practices in the context<br>Medical History<br>Vaccination<br>Health care-seeking knowledge, attitude, behaviour<br>Family planning variables<br>Others
Maternal & Pregnancy-related	Gestational age<br>Intra Uterine Growth Restriction<br>Conditions/ Diseases in pregnancy<br>Urinary Tract Infections<br>Mode and Place of delivery<br>Skilled birth attendance<br>Foul-smelling liquor<br>Prolonged rupture of membranes

For peer review only - http://bmjopen.bmj.com/site/about/guidelines.xhtml

	Prolonged labour<br>Intrapartum fever<br>Others
Environment-related	Place of residence (e.g. urban, rural, peri-urban)<br>Housing<br>Overcrowding<br>Water, sanitation and hygiene<br>Indoor Air Pollution (including cooking smoke, mosquito coils/ repellents, Second-hand smoke exposure)<br>Pets/ Livestock<br>Rainfall<br>Outdoor Air pollution<br>Seasonality<br>Others
Health System – related	
Iatrogenic	

Search methods for identification of studies

An exhaustive list of search terms will be used to develop a sensitive search strategy for final searches. There will be no restrictions on the time period for database searches, as studies published at any time are eligible for inclusion.

Electronic searches will be done on nine databases: PubMed, Ovid MEDLINE, Web of Science, CINAHL, EMBASE, SCOPUS, IMSEAR, ProQuest and IndMED. Handsearching will be performed for those journal volumes/ issues that are not available in electronic searches and for conference proceedings to review the references and contact the authors for full texts of identified literature. Dissertations/ thesis will be identified on Shodhganga (INFLIBNET), and reports/ fact sheets will be retrieved by searching national websites. Snowballing will be done to identify relevant studies from reference lists of included studies and systematic reviews. However, such systematic reviews will not be eligible for inclusion in our review. Moreover, subject experts and authors of identified studies/organizations will be contacted to procure relevant studies for inclusion in the review.

Table 2 contains an example of our search strategy.

Table 2. Search strategy for PubMed.

Strategy: #1 AND #2 AND #3	
#1	(((Neonate* OR childhood OR neonatal* OR newborn* OR "young infant" OR child OR pediatric* OR paediatric* OR "neonatal period" OR infant* OR "newborn infant"))

For peer review only - http://bmjopen.bmj.com/site/about/guidelines.xhtml

#2	((((((((((((((((((((((((((((((Pneumonia*) OR Pneumon*) OR "community acquired pneumonia") OR "congenital pneumonia") OR "hospital acquired pneumonia") OR "nosocomial pneumonia") OR "ventilator associated pneumonia") OR "early onset pneumonia") OR "late onset pneumonia") OR "infective pneumonia") OR "infectious pneumonia") OR "meconium aspiration syndrome") OR "meconium aspiration") OR "lipoid pneumonia") OR sepsis*) OR "acute respiratory infections") OR "early onset sepsis") OR "chemical pneumonia") OR "aspiration pneumonia") OR "late onset sepsis") OR infection*) OR "nosocomial infection") OR "early onset infection") OR "late onset infection") OR "acute lower respiratory infection") OR "hospital acquired infection") OR "congenital infection") OR "viral pneumonia") OR "gastro esophageal reflux disease") OR "cystic fibrosis")
#3	((("Risk factor" OR determinant* OR risk* OR predictor* OR "relative risk" OR "odds ratio" OR "attributable risk" OR "population attributable fraction"))))
Geographical Filter: India<br>Language Filter: English	

Data collection and management

Endnote (v. x7) will be used to manage the search results and perform screening. The data extraction will be performed on Microsoft Excel 2007, and STATA (v.13) will be used for statistical analysis.

Selection of studies

Screening of search results for eligible studies will be performed by two authors (MG and SM) independently in title, abstract and full-text stages. During the title screening stage, titles approved by either of the authors will proceed to abstract screening. At the abstract screening stage, the abstracts that are approved by either of the authors will proceed to full-text screening. Abstracts rejected by both authors will be excluded. During full-text screening, those records that are approved by both the authors will be included in our review. Disagreements will be resolved through consensus with an arbitrator (TL) and/or senior review authors (SN and LL). A PRISMA chart will be used to illustrate the study selection process.[24]

For peer review only - http://bmjopen.bmj.com/site/about/guidelines.xhtml

Data extraction

Data extraction will be performed on pilot-tested spreadsheet by two authors (MG and SM) independently. Disagreements between authors will be resolved by arbitrated consensus. The data extraction form has been provided in the Supplementary File, and was developed in consultation with subject and methodological experts. Pilot-testing of the data extraction form was performed on different study designs to ensure that it is complete and adequate enough for various study designs. Data will be captured for study details, methodological characteristics, risk factors and determinants and their measure and strength of association and other information (e.g. funding, limitations, conflict of interest, etc).

Dealing with missing data

Authors of studies with missing data or methodological information will be contacted by email to obtain the required data/information. If missing data cannot be retrieved despite this effort, the particular study will be excluded from the meta-analysis, and a narrative summary will discuss the findings on risk factors/ determinants from the study.

Risk of bias assessment

Risk of bias assessment will be conducted two authors (SM and TL) independently, at the study level. Checklists will be based on the design of the study as follows:

- Case control study: Newcastle-Ottawa Scale (NOS)[25]
- Cohort study: Newcastle-Ottawa Scale (NOS)[25]
- Cross-sectional study: an adapted version of NOS[26]
- Case series: Institute of Health Economics (IHE) criteria[27]

Disagreements will be resolved through discussion and consensus in the presence of a third (MG) or senior author(s) (SN and LL).

Data analysis

A meta-analysis will be performed using a random-effects model. The I^2 statistic will be calculated and reported. Categorical data will be summarized using odds ratio (OR) and/or relative risk (RR). Continuous data will be summarized using the standardized mean

For peer review only - http://bmjopen.bmj.com/site/about/guidelines.xhtml

difference (SMD). The summary measures will be pooled together based on the study design. A forest plot will be generated and pooled estimates will be reported with 95% confidence intervals.[28]

Based on the availability of data, a subgroup analysis will be conducted according, but not limited, to study design, study setting, the type of neonatal pneumonia, and the timing of onset of pneumonia among neonates. A sensitivity analysis will be performed to determine the robustness of the findings, by removing one study at a time and noting the change in pooled estimate. Reporting bias will be assessed if there are more than ten studies that are included in our review. A funnel plot will be generated and Egger's test performed to assess the degree of asymmetry. Based on the data availability, attempts will be made to perform a meta-regression to assess covariates' effect on the pooled estimate, and adjust for this effect. The characteristics and findings of the review will be presented in the form of tables and a narrative summary. Additionally, the narrative summary will discuss the impact of study quality and limitations of included studies on the findings.

Synthesis of project findings: The analysis plan for the collation of the findings from the three reviews and the qualitative study will be described elsewhere.

Quality assessment of the systematic review and meta-analysis: "Assessing the Methodological quality of Systematic Reviews" (AMSTAR) criteria will be used to evaluate the systematic review's methodological rigor.[29] The "Grading of Recommendations Assessment, Development and Evaluation" (GRADE) approach will be used to assess the quality of evidence generated, and a summary of findings (SoF) table will be generated and reported using GRADEPro.

Ethics and dissemination:

Ethics: The protocol of the entire project consisting of three reviews, a qualitative study and data triangulation has been approved by the host institution's ethics body, (Institutional Ethics Committee at Manipal University in Manipal, India), and the 'Health Ministry Screening Committee' (HMSC) under the Ministry of Health and Family Welfare, Government of India.

For peer review only - http://bmjopen.bmj.com/site/about/guidelines.xhtml

Dissemination: The study findings will be disseminated among relevant stakeholders through knowledge dissemination workshops, publications, plain language summaries, policy brief(s) etc.

Reporting of the systematic review and meta-analysis: The PRISMA [16] and MOOSE guidelines [24] will be used to report our review.

Acknowledgements

We thank the following persons for their guidance and support throughout our protocol development process: Dr. Manoj Das, Director Projects, The INCLEN Trust International, New Delhi; Dr Anju Sinha, Deputy Director General, Scientist 'E', Division of Child Health, Indian Council of Medical Research, New Delhi; Dr. K.K. Diwakar, Professor and Head, Department of Neonatology, Associate Dean, Malankara Orthodox Syrian Church Medical College, Kerala; Mrs. Ratheebhai V., Senior Librarian and Information Scientist, at Manipal School at Communication, Manipal University, Manipal; Dr. Ravinder M. Pandey, Professor and Head, Department of Biostatistics, All India Institute of Medical Sciences, New Delhi; Dr. B. Shantharam Baliga, Professor, Department of Paediatrics, Kasturba medical college, Mangalore, Karnataka, ; Dr. Shirish Darak, Senior researcher, PRAYAS, Pune, Maharashtra; Dr. Unnikrishnan B., Associate Dean and Professor, Department of Community Medicine, Kasturba Medical College, Mangalore. We also thank Public Health Evidence South Asia (PHESA), Manipal University, for providing institutional and infrastructural support. We thank The INCLEN Trust International, New Delhi, and The Bill and Melinda Gates Foundation for their financial support.

Contributorship statement: SN is the guarantor of the review. SN, BV and LL conceived the research idea and reviewed the manuscript. SN and LL provided overall technical guidance. In addition, LL assisted in developing search terms. MG, SM and TL designed the protocol, drafted the manuscript, and developed and pilot tested the search strategies and data extraction form.

Funding: This project is supported by a grant from Bill & Melinda Gates Foundation [grant OPP1084307] to The INCLEN Trust International and sub-grant to Manipal University

For peer review only - http://bmjopen.bmj.com/site/about/guidelines.xhtml

For peer review only

[subgrant INC2015GNT004). The views expressed through this project do not necessarily represent the views of Bill and Melinda Gates Foundation or The INCLEN Trust International or Manipal University.

Data sharing statement: All data supporting this study will be provided as supplementary material together with the manuscript of the study's final results.

Competing Interests: All authors have completed the ICMJE uniform disclosure form at www.icmje.org/coi_disclosure.pdf and declare: all authors had financial support (grants) from Bill and Melinda Gates Foundation (grant OPP1084307) to The INCLEN Trust International and sub-grant to Manipal University (subgrant INC2015GNT004)., during the conduct of the study and for the submitted work; no financial relationships with any organisations that might have an interest in the submitted work in the previous three years; no other relationships or activities that could appear to have influenced the submitted work.

References

1. Bennett D, Bisanzio D, Deribew A, et al. Global, regional, and national under-5 mortality, adult mortality, age-specific mortality, and life expectancy, 1970–2016: a systematic analysis for the Global Burden of Disease Study 2016. 2017
2. Department of Health & Family Welfare MoHFW, Government of India. Ch 5: Child Health Program, Annual Report 2014-15 2015 [cited 2016 18/1/2016]. Available from: http://www.mohfw.nic.in/WriteReadData/l892s/5658789632541236.pdf.
3. World Health Organisation (WHO). Neonatal and Child Health Profile, India: Department of Maternal Newborn Child and Adolescent Health 2013 [cited 2016 19/1/2016]. Available from: http://www.who.int/maternal_child_adolescent/epidemiology/profiles/neonatal_child/ind.pdf.
4. Reiterer F. Neonatal pneumonia. *Neonatal Bacterial Infection InTech* 2013
5. PHFI A. SC-State of India's Newborns (SOIN) 2014 - a report. *Public Health Foundation of India, All India Institute of Medical Sciences and Save the Children New Delhi, India Available at http://wwwnewbornwhoccorg/SOIN_PRINTED* 2014:9-2014.
6. UNICEF. Infant and Child mortality in India: Levels, trends and determinants (Fact sheet) [cited 2016 3/9/2016]. Available from: http://unicef.in/CkEditor/ck_Uploaded_Images/img_1364.pdf.
7. Dennehy P. Respiratory infections in the newborn. *Clinics in perinatology* 1987;14(3):667-82.
8. Webber S, Wilkinson A, Lindsell D, et al. Neonatal pneumonia. *Archives of disease in childhood* 1990;65(2):207-11.
9. Duke T. Neonatal pneumonia in developing countries. *Archives of Disease in Childhood-Fetal and Neonatal Edition* 2005;90(3):F211-FF19.
10. Petdachai W. Ventilator-associated pneumonia in a newborn intensive care unit. *Southeast Asian journal of tropical medicine and public health* 2004;35:724-29.

For peer review only - http://bmjopen.bmj.com/site/about/guidelines.xhtml

11. Balakrishnan S. Lipoid pneumonia in infants and children in South India. *Br med J* 1973;4(5888):329-31.
12. Ghimire M, Bhattacharya S, Narain J. Pneumonia in South-East Asia Region: Public health perspective. *The Indian journal of medical research* 2012;135(4):459.
13. Nair NS, Lewis LE, Lakiang T, et al. Factors associated with mortality due to neonatal pneumonia in India: a protocol for systematic review and planned meta-analysis. *BMJ open* 2017;7(9):e017616.
14. Nair NS, Lewis LE, Murthy S, et al. Treatment options and barriers to case management of neonatal pneumonia in India: a protocol for a scoping review. *BMJ open* 2017;7(9):e017617.
15. Nair NS, Lewis LE, Lakiang T, et al. Risk factors and barriers to case management of neonatal pneumonia: protocol for a pan-India qualitative study of stakeholder perceptions. *BMJ open* 2017;7(9):e017403.
16. Stroup DF, Berlin JA, Morton SC, et al. Meta-analysis of observational studies in epidemiology: a proposal for reporting. *Jama* 2000;283(15):2008-12.
17. Shamseer L, Moher D, Clarke M, et al. Preferred reporting items for systematic review and meta-analysis protocols (PRISMA-P) 2015: elaboration and explanation. *Bmj* 2015;349:g7647.
18. WHO. Risk Factors [cited 2016 19/1/2016].
19. Whitehead M, Dahlgren G. What can be done about inequalities in health? *The lancet* 1991;338(8774):1059-63.
20. Sonego M, Pellegrin MC, Becker G, et al. Risk Factors for Mortality from Acute Lower Respiratory Infections (ALRI) in Children under Five Years of Age in Low and Middle-Income Countries: A Systematic Review and Meta-Analysis of Observational Studies. *PLoS ONE* 2015;10(1):e0116380. doi: 10.1371/journal.pone.0116380
21. Wonodi CB, Deloria-Knoll M, Feikin DR, et al. Evaluation of risk factors for severe pneumonia in children: the Pneumonia Etiology Research for Child Health study. *Clinical infectious diseases* 2012;54(suppl 2):S124-S31.
22. Patwari A. Risk factors for mortality in children hospitalized with Pneumonia. *Indian pediatrics* 2012;49(11):869.
23. Shah SI, Zemichael O, Meng HD. Predictors of mortality, length of stay and co-morbid hypothermia in hospitalized neonates with pneumonia in Eritrea, Africa. *Journal of tropical pediatrics* 2012;58(3):238-41.
24. Liberati A, Altman DG, Tetzlaff J, et al. The PRISMA statement for reporting systematic reviews and meta-analyses of studies that evaluate health care interventions: explanation and elaboration. *Annals of internal medicine* 2009;151(4):W-65-W-94.
25. Wells G, Shea B, O'connell D, et al. The Newcastle-Ottawa Scale (NOS) for assessing the quality of nonrandomised studies in meta-analyses, 2000.
26. Herzog R, Álvarez-Pasquin MJ, Díaz C, et al. Are healthcare workers' intentions to vaccinate related to their knowledge, beliefs and attitudes? A systematic review. *BMC public health Available at: http://static-contentspringercom/esm/art%3A101186%2F1471-2458-13-154/MediaObjects/12889_2012_5111_MOESM3_ESMdoc* 2013;13(1):1.
27. Moga C, Guo B, Schopflocher D, et al. Development of a quality appraisal tool for case series studies using a modified Delphi technique. Institute of Health Economics. Alberta Canada, 2012.
28. DerSimonian R, Laird N. Meta-analysis in clinical trials revisited. *Contemporary clinical trials* 2015;45:139-45.
29. Shea BJ, Grimshaw JM, Wells GA, et al. Development of AMSTAR: a measurement tool to assess the methodological quality of systematic reviews. *BMC medical research methodology* 2007;7(1):1.

For peer review only - http://bmjopen.bmj.com/site/about/guidelines.xhtml

For peer review only

DRAFT DATA EXTRACTION FORM

Section 1: General Information	Serial No	
	Date of extraction	*dd/mm/yyyy*
	Name of review author completing this form	*First name/middle name/ family name*
	Date form completed	*dd/mm/yyyy*
	Name of review author checking the data extracted to this form	*First name/middle name/ family name*
	Name of review author checking the data extracted to this form	*First name/middle name/ family name*
	Citation	*Vancouver style*
	URL	
	Other information and notes	
	Study ID	
Section 2: ELIGIBILITY	Study is reported in the Indian context?	*Yes/ No*
	Study is of observational design?	*Yes/ No*
	Study reports risk factor quantitative analysis for pneumonia in neonates?	*Yes/ No*
	Is study eligible for inclusion?	*Yes/ No*
Section 3: AUTHOR INFORMATION	Corresponding Author contact details for study	*Name*
		Affiliation
		Email id
		Phone number (if available)
	Further information required	
	Correspondence with authors successful or not; what information was received and when	
	Notes (Unpublished – for own use)	
Section 4: METHODS AND METHODOLOGY	Aim of study (as reported)	
	Study Design	
	Geographic location (s)	
	Setting (s)	*Hospital/ community*
		rural/ urban/ semi-urban
	Study Duration	
	Start date/ year	
	End date/ year	
	Number of groups	
	Inclusion criteria (for each group)	
	Exclusion criteria (for each group)	
	Operational definition of pneumonia used for study	
	Number in each group	
	Total subjects	
	Non-respondents	
	Duration of participation	*Recruitment*
		Data collection
		Follow up

For peer review only

	Method of recruitment of participants (e.g. phone, mail, clinic)	
	Data collection method	
	Source(s) of data for each group	
	Assumptions made	
	Variables included	*Independent*
		Dependant
	Confounding factors accounted for	*Confounding*
	Statistical methods	*Software*
		Tests
	Ethical approval obtained?	
	Informed consent obtained?	
	Sampling Technique	
	Sampling strategy	
	Sample size calculation:	
	Sample size	
	Eligible for inclusion	
	Excluded	
	Refused to take part	
	Cases/study cohort	
	Controls	
	Excluded (for each group; with reasons if relevant)	
	Withdrawn (for each group; with reasons if relevant)	
	Lost to follow up (for each group; with reasons)	
	Included in the analysis (for each group, for each outcome)	
**Section 5: RESULTS**	Population characteristics	*Age (years)*
		Gender
		Educational
		Income
		Socioeconomic strata
	Incidence/ Prevalence	*Number*
		Proportion
		other
		time to onset
	Risk factor	
	Group A events	
	Group A total	
	Group A rate and estimate	
	p	
	Confidence Interval (CI)	*lower*
		upper
	Group B events	
	Group B total	
	Group B rate and estimate	

For peer review only

	p	
	Confidence Interval (CI)	*lower*
		upper
	Group A Mean	
	Group A SD	
	Group B Mean	
	Group B SD	
	p	
	CI	*lower*
		upper
	Other findings	
	Conclusions	
	Limitations	
	Funding	

For peer review only

PRISMA-P (Preferred Reporting Items for Systematic review and Meta-Analysis Protocols) 2015 checklist: recommended items to address in a systematic review protocol*

Section and topic	Item No	Checklist item	Compliance
**ADMINISTRATIVE INFORMATION**			
Title:			
Identification	1a	Identify the report as a protocol of a systematic review	Pg 1& 2
Update	1b	If the protocol is for an update of a previous systematic review, identify as such	Not applicable
Registration	2	If registered, provide the name of the registry (such as PROSPERO) and registration number	Pg 1
Authors:			
Contact	3a	Provide name, institutional affiliation, e-mail address of all protocol authors; provide physical mailing address of corresponding author	Pg 1
Contributions	3b	Describe contributions of protocol authors and identify the guarantor of the review	Pg 9
Amendments	4	If the protocol represents an amendment of a previously completed or published protocol, identify as such and list changes; otherwise, state plan for documenting important protocol amendments	Not applicable
Support:			
Sources	5a	Indicate sources of financial or other support for the review	Pg 9
Sponsor	5b	Provide name for the review funder and/or sponsor	Pg 9
Role of sponsor or funder	5c	Describe roles of funder(s), sponsor(s), and/or institution(s), if any, in developing the protocol	Pg 9
**INTRODUCTION**			
Rationale	6	Describe the rationale for the review in the context of what is already known	Pg 3
Objectives	7	Provide an explicit statement of the question(s) the review will address with reference to participants, interventions, comparators, and outcomes (PICO)	Pg 3 Modified accordingly for review of non-interventional studies
**METHODS**			
Eligibility criteria	8	Specify the study characteristics (such as PICO, study design, setting, time frame) and report characteristics (such as years considered, language, publication status) to be used as criteria for eligibility for the review	Pg 4 Modified accordingly for

For peer review only

			review of non-interventional studies
Information sources	9	Describe all intended information sources (such as electronic databases, contact with study authors, trial registers or other grey literature sources) with planned dates of coverage	Pg 4-5
Search strategy	10	Present draft of search strategy to be used for at least one electronic database, including planned limits, such that it could be repeated	Pg 5
Study records:			
Data management	11a	Describe the mechanism(s) that will be used to manage records and data throughout the review	Pg 5
Selection process	11b	State the process that will be used for selecting studies (such as two independent reviewers) through each phase of the review (that is, screening, eligibility and inclusion in meta-analysis)	Pg 5-6
Data collection process	11c	Describe planned method of extracting data from reports (such as piloting forms, done independently, in duplicate), any processes for obtaining and confirming data from investigators	Pg 6
Data items	12	List and define all variables for which data will be sought (such as PICO items, funding sources), any pre-planned data assumptions and simplifications	Pg 6
Outcomes and prioritization	13	List and define all outcomes for which data will be sought, including prioritization of main and additional outcomes, with rationale	Pg 7
Risk of bias in individual studies	14	Describe anticipated methods for assessing risk of bias of individual studies, including whether this will be done at the outcome or study level, or both; state how this information will be used in data synthesis	Pg 6-7
Data synthesis	15a	Describe criteria under which study data will be quantitatively synthesised	Pg 7
	15b	If data are appropriate for quantitative synthesis, describe planned summary measures, methods of handling data and methods of combining data from studies, including any planned exploration of consistency (such as I^2, Kendall's τ)	Pg 7
	15c	Describe any proposed additional analyses (such as sensitivity or subgroup analyses, meta-regression)	Pg 7
	15d	If quantitative synthesis is not appropriate, describe the type of summary planned	Pg 7
Meta-bias(es)	16	Specify any planned assessment of meta-bias(es) (such as publication bias across studies, selective reporting within studies)	Pg 7-8
Confidence in cumulative evidence	17	Describe how the strength of the body of evidence will be assessed (such as GRADE)	Page 8

*** It is strongly recommended that this checklist be read in conjunction with the PRISMA-P Explanation and Elaboration (cite when available) for important clarification on the items. Amendments to a review protocol should be tracked and dated. The copyright for PRISMA-P (including checklist) is held by the PRISMA-P Group and is distributed under a Creative Commons Attribution Licence 4.0.**

From: Shamseer L, Moher D, Clarke M, Ghersi D, Liberati A, Petticrew M, Shekelle P, Stewart L, PRISMA-P Group. Preferred reporting items for systematic review and meta-analysis protocols (PRISMA-P) 2015: elaboration and explanation. BMJ. 2015 Jan 2;349(jan02 1):g7647.

For peer review only
